# Association of Genetic Polymorphisms in FOXA1 with the Progression of Genetic Susceptibility to Gastric Cancer

**DOI:** 10.1155/2020/3075837

**Published:** 2020-01-27

**Authors:** Guo-wen Ding, Xu-yu Gu, Zhe Dai, Hui-wen Pan, Xiao-yan Wang, Heng Zhang, Yu Fan

**Affiliations:** ^1^Department of Thoracic Surgery, Affiliated People's Hospital of Jiangsu University, Zhenjiang, Jiangsu 212002, China; ^2^Institute of Oncology, Affiliated People's Hospital of Jiangsu University, Zhenjiang, Jiangsu 212002, China; ^3^Department of Gastroenterology, The First People's Hospital of Suqian, Jiangsu Province, China; ^4^Department of General Surgery, Nanjing Lishui District People's Hospital, Zhongda Hospital Lishui Branch, Southeast University, Nanjing 211200, China

## Abstract

**Objective:**

To investigate the relationship between polymorphism of FOXA1 gene rs12894364 and rs7144658 and susceptibility to gastric cancer.

**Methods:**

A case-control study was conducted to select 577 cases of primary gastric cancer and 678 cases of normal control. We extracted whole blood genomic DNA and amplified the target gene fragment by PCR. The genotyping and allele was tested through a snapshot method.

**Results:**

There was no significant difference in the frequency distribution of genotype between the case group and control group (*P* > 0.05). Stratified analyses showed the SNPs were not correlated with the susceptibility of GC according to different age, gender, cigarette smoking, and alcohol drinking status.

**Conclusion:**

There is no significant correlation between the polymorphisms of FOXA1 gene rs12894364 and rs7144658 and the risk of gastric cancer.

## 1. Introduction

Gastric cancer is one of the most common malignant tumors in China. Due to its insidious onset and the current lack of effective early diagnostic molecular markers, patients often found to be advanced, resulting in a 5-year survival rate < 25% [1, 2]. Therefore, finding important molecules involved in the development of gastric cancer and investigating their mechanisms are of great significance for early diagnosis and treatment of gastric cancer. With the development of the third generation of genetic marker technology, the research and exploration of the pathogenesis of gastric cancer has been further developed. Single nucleotide polymorphism (SNP) analysis is one of the most important methods of studying complex diseases and genetic recognition of populations. The study of tumor-associated gene polymorphisms may provide new predictors and intervention targets for cancer therapy.

The forkhead box proteins (Fox) are highly conserved in evolution. Each member has a forkhead frame (or pterygoid) DNA-binding domain of about 110 amino acids in length. The hepatocyte nuclear factor 3 alpha (HNF3a) is the first member of the forkhead family of proteins found in mammals. It is named FoxAl [3] that is required for individual growth and development. It is expressed in endoderm (such as the liver, lung, pancreas), mesoderm (such as the kidney, uterus, breast), and ectoderm (such as brain, olfactory epithelium) in adult tissues, and plays different functions [4]. What is more, FoxAl has a close relationship with the occurrence and progression of a variety of clinical tumors [5–8]. However, the role of FoxAl in the development of gastric cancer is not yet clear. The levels of FOXA1 protein and mRNA in gastric cancer tissues were significantly higher than those in adjacent tumor tissues. In addition, clinical association analysis showed that positive FOXA1 expression was associated with poor clinicopathological features in patients with gastric cancer, including poor tumor differentiation, large tumor size, and advanced stage of lymph node metastasis. Notably, the 5-year overall and relapse-free survival of gastric cancer patients with FOXA1 positive expression was poor. In vivo studies showed that FOXA1 knockdown significantly inhibited tumor growth in gastric cancer in nude mouse xenograft models. Therefore, FOXA1 can be a promising prognostic indicator and an attractive therapeutic target for gastric cancer. The clinical significance of FOXA1 and its biological function in gastric cancer remains unknown. Therefore, this present study used a case-control method to compare the genotypes and alleles of FOXA1 gene rs12894364 and rs7144658 in gastric cancer patients and healthy controls to analyze the relationship between FOXA1 gene polymorphism and gastric cancer susceptibility. At the same time, combined with the patient's clinical parameters, such as gender, age, smoking history, and drinking history, we can analyze the correlation between them comprehensively by providing a theoretical basis for early screening and early treatment of gastric cancer.

## 2. Research Object and Method

Totally, 577 consecutive GC patients and 678 cancer-free controls were recruited from the Affiliated People's Hospital of Jiangsu University, between May 2013 and June 2017 as described previously. The present study was approved by the Ethics Committee of Affiliated People's Hospital of Jiangsu University. Written informed consent was obtained from patients and controls. Clinical data of patients were obtained from questionnaires and medical records. Peripheral blood (2 ml) was collected from each subject. DNA was extracted from the peripheral blood according to the instructions. The PCR amplification products were purified by ExoI and FastAP, and then extended. After the extension reaction, the ABI3730XL was used for sequencing to detect genotyping. The gene polymorphism was detected by a Snapshot method and the 5% samples were randomly selected for reinspection to ensure the accuracy of the test results.

The random sampling method was used to select GC group samples. The sample content was estimated by using the sample power software, and the minor allele frequency (MAF) was selected to be greater than 5%, the variation genotype frequency was about 8% or more, the accuracy of statistical test was 80%, and the two-sided test significance level at time *α* = 0.05. According to the power and sample size calculation software, the odds ratio (OR) was about 1.23/0.81. The randomly selected GC sample size in this study conforms to the requirements.

In this study, a case-control study was conducted and a logistics regression model was applied to analyze the impact of one or more causes on the outcome of a classification, and a key indicator OR was calculated. In univariate and multivariate analyses, check for correlations between FOXA1 and other variables. The independent effects of gender, age, smoking, and alcohol consumption were examined separately, and the effects of stratification were examined separately for gender, age, smoking, alcohol consumption, and the effects of stratification among the groups.

## 3. Statistical Method

SPSS 20.0 (SPSS Inc., Chicago, IL, USA) was used for data analysis. A chi-square analysis test was used to test whether the distributions of polymorphisms in cases and controls fit the Hardy-Weinberg equilibrium. We use logistic regression to calculate the risk of GC attributed to SNP genotypes and alleles.

## 4. Results

Table 1 shows that rs12894364 and rs7144658 are located in 14th chromosome. Their category is protein coding. The minor allele frequency (MAF) of rs12894364 in our controls is 0.119. The Hardy-Weinberg equilibrium test in our controls is 0.659 (*P* > 0.05), which means that our sample population is representative. We use the Snapshot method as genotyping and the percentage of the successful tests is 98.73%. The minor allele frequency (MAF) of rs7144658 for Chinese in genecard database is 0.121 and in our controls is 0.116. The Hardy-Weinberg equilibrium test in our controls is 0.940 (*P* > 0.05), which means that our sample population is representative. We use the Snapshot method as genotyping and the percentage of the successful tests is 98.65%.

The results in Table 2 showed the characteristics of the study subjects, including demographics and environmental risk factors. Smoking rate was much higher in the case group as compared with the control group (34.49% vs. 27.29%, *P* = 0.006). The demographics (age and sex) was well matched (*P* = 0.635 and *P* = 0.698, respectively; Table 2). That indicated the occurrence and development of smoking and gastric cancer. Of the alcohol consumption, no significant difference was observed between GC patients and controls (*P* = 0.443, Table 2).

The frequency distribution and logistic regression analysis of the FOXA1 gene rs12894364 polymorphism in gastric cancer and the control group showed that with reference to wild-type CC, the frequency distribution of TC heterozygous mutations was not statistically significant between the two groups (*P* = 0.668) and there was no statistical difference in gender, age, smoking, and alcohol consumption after logistic regression adjustment (*P* = 0.796); the frequency distribution of TT homozygous mutants was also not statistically significant (*P* = 0.649), and there was no statistical difference after logistic regression adjustment (*P* = 0.874). In the dominant model, the frequency distribution of TC+TT mutations was not statistically significant in the case-control group (*P* = 0.758), and the difference was not statistically significant after regression adjustment (*P* = 0.858). In the recessive model, the frequency distribution was not statistically different (*P* = 0.776). According to gender, age, smoking, and drinking, after logistic regression analysis, there was still no statistical difference between the two groups (*P* = 0.810) (Table 3).

The results of the frequency distribution and logistic regression analysis of FOXA1 rs7144658 in gastric cancer and the control group showed that the difference between the two groups with the wild-type TT as the reference type was not statistically significant (*P* = 0.720), and the difference was not statistically significant after logistic regression analysis adjusted according to gender, age, smoking, and drinking (*P* = 0.641). Also, there was no statistically significant difference in the frequency distribution of CC homozygous mutant (*P* = 0.916), and there was no statistically significant difference after logistic regression analysis (*P* = 0.882). In the distribution of the dominant model\recessive model, there was no statistical difference (*P* = 0.713). According to logistic regression analysis, there was still no statistical difference (*P* = 0.631; 0.915) (Table 4).

Compared with the frequency distribution of C allele in rs12894364, the T allele was higher in the case group than in the control group (12.57% > 11.92%), and the difference was not statistically significant (*P* < 0.628). The frequency distribution of rs7144658 allele was not statistically significant in the case-control group (*P* = 0.721) (Table 5).

According to stratification results, the polymorphism of FOXA1 rs12894364 showed that with wild-type CC as a reference genotype, wild-type TC, homozygous TT, dominant model, and the recessive model in the female group were not statistically significant (*P* = 0.288, *P* = 0.795, *P* = 0.280, *P* = 0.846).There were no significant differences in wild-type TC, homozygous TT, dominant models, and recessive models among the gender, smoking, or drinking groups (Table 6).

The stratified results of FOXA1 rs7144658 polymorphism showed that taking the wild-type TT as the reference genotype, we found that the wild-type TC, homozygous CC, dominant model, and recessive model had no statistical significance in the female group (*P* = 0.282, *P* = 0.543, *P* = 0.242, *P* = 0.585). There were no statistically significant differences in wild-type TC, homozygous CC, dominant model, or recessive model in the gender, smoking, or drinking group (Table 7).

## 5. Discussion

Gastric cancer is a multifactorial disease including diet, genetic factors, environmental factors, immune factors, infections, and inflammation. These factors inevitably lead to the imbalance of some signaling pathways, which are closely related to the growth and regulation factors of gastric cancer. The occurrence and progression of tumors are inseparable from the regulation of transcription factors. The FOXA family is closely related to the occurrence, proliferation, invasion, and metastasis of malignant tumors. FOXA1 is an important member of the FOX family, widely distributed over the body. FOXA1 binds to the promoter region of the target gene chromosome, leading to the restructuring of nucleosome structure, which promotes the binding of other transcription factors to the promoter region of the target gene and promotes the transcription of the target gene. Therefore, FOXA1 plays an important role in various biological processes such as organ development, body metabolism, and tumorigenesis [9]. Studies in prostate cancer, thyroid cancer, and glioma have shown that FOXA1 is highly expressed in tumor tissues, has a significant correlation with tumor grade, invasion, and metastasis and poor prognosis of patients, and plays a role in promoting tumorigenesis and development [10–12]. However, in the study of breast cancer and endometrial cancer, it was found that the positive expression of FOXA1 was significantly negatively correlated with the poor prognosis of patients, which plays a role in inhibiting the development of tumors [13, 14]. The above results indicate that the role of FOXA1 in the occurrence and development of tumors is specific. In different tumor tissues, FOXA1 can play a role in promoting or inhibiting the development of tumors.

However, the biological function of FOXA1 in gastric cancer tissues is still unclear. A significant increase in mRNA and protein levels of FOXA1 was observed in gastric cancer tissues compared to adjacent tumor tissues. Moreover, it is more important to reveal that the positive expression of FOXA1 is associated with poor clinicopathological features and poor prognosis in patients with gastric cancer. Therefore, FOXA1 is expected to be a novel biomarker with significant value in predicting the clinical outcome of gastric cancer patients. The potential carcinogenic role of FOXA1 in gastric cancer has led us to investigate its biological role. Previous studies [15, 16] have confirmed that FOXA1 is a forkhead transcription factor regulating chromatin structure and recruiting other transcription factors to facilitate downstream target transcription. Functionally, FOXA1 is an important regulator of cell proliferation, cell cycle, and apoptosis. Both in vitro and in vivo studies have demonstrated that FOXA1 inhibition can inhibit the proliferation and induce apoptosis of gastric cancer cells [17–19]. Therefore, our data suggest that FOXA1 plays a carcinogenic role in gastric cancer by promoting cell proliferation and preventing apoptosis. The Hippo-YAP signaling pathway has been found to play a key role in gastric cancer. The expression of YAP has been confirmed to be significantly higher than that of previous studies consistent with normal gastric mucosa [20–23]. YAP regulates proliferation and apoptosis of gastric cancer cells [24–26]. Therefore, YAP is regarded as a therapeutic target for gastric cancer. Interestingly, recent studies on hepatocellular carcinoma have shown that FOXA1 can open up the dense chromatin surrounding the CREB binding site in the YAP promoter and promote CREB-mediated YAP transcription, leading to increased expression of YAP in HCC cells [27]. Therefore, we hypothesized that FOXA1 might regulate the proliferation and apoptosis of gastric cancer cells by regulating the expression of YAP [21]. FOXA1 mRNA and protein levels were significantly reduced by foxa1-specific shRNA inhibition of FOXA1 expression in gastric cancer cells. These results suggest that FOXA1 may regulate cell proliferation and apoptosis at least in part by regulating the expression of YAP in gastric cancer cells. Genomic profiles of targeted therapies and GC are also targeted, but their biological effects are still partially obscured. At present, most studies on the FOXA1 gene polymorphism revolve around type 2 diabetes and breast cancer [28, 29]. Zhang et al. [30] and other studies found that the FOXA1 gene rs4442975 locus was not associated with breast cancer risk in the Chinese population, and negative results were also observed in all subgroups of ER, PR, smoking, drinking, and menopausal stratification. There is no research on the relationship between FOXA1 gene polymorphism and gastric cancer.

Based on the above background, the polymorphisms of FOXA1 gene rs12894364 and rs7144658 in the primary gastric cancer group and the control group were detected in this study. The genotype frequency distribution at the site was consistent with the Hardy-Weinberg equilibrium law in the control group, and the sample was still considered to have good population representativeness. Using the association analysis of case-control studies, it was found that the allele frequencies of rs12894364 and rs7144658 were not statistically different between the case group and the control group. Comparing its genotype frequency distribution and gene model, there was also no statistical significance between the case group and the control group. This result does not prove that the FOXA1 gene is a susceptibility gene for gastric cancer. Our findings show that FOXA1 rs12894364 and rs7144658 polymorphisms were not implicated with altered susceptibility of GC in different age, gender, cigarette smoking, and alcohol drinking status.

The negative results obtained in this study may be affected by the following factors: small sample content, insufficient genetic marker sites, and the presence of other biases. Stomach cancer is caused by a combination of environmental factors and the accumulation of specific genetic changes. Dietary factors play an important role in the development of gastric cancer, especially in the case of intestinal adenocarcinoma. In addition, due to the heterogeneity of the tumor, different pathological types and stages of gastric cancer may also lead to the deviation of the results. Gastric cancer is divided into intestinal type, diffuse type, and mixed type according to Lauren type, and FOXA1 expression state is different in different types of gastric cancer. Meanwhile, Epstein-Barr virus (EBV) infection of HP is a risk factor for gastric cancer [31, 32]. Therefore, the results may have certain limitations so that the association between FOXA1 gene and gastric cancer cannot be completely ruled out. In addition, considering gene-gene interactions and gene environment interactions in the pathogenesis of many diseases, especially chronic diseases [33], the FOXA1 gene polymorphism may be related to other gene polymorphisms or environmental factors. This interaction affects the incidence of gastric cancer in humans. It is proposed to analyze gene-gene interactions and gene-environment interactions in further research in the future, providing further evidence for the pathogenesis of gastric cancer.

## Figures and Tables

**Table 1:** Primary information for gene FOXA1 gene rs12894364 and rs7144658 polymorphisms.

Genotyped SNPs	Gene	Chr Pos (NCBI Build 38)	Category	MAF^a^ for Chinese in database	MAF in our controls (*n* = 678)	*P* value for the HWE^b^ test in our controls	Genotyping method	Genotyping value (%)
rs12894364	FOXA1	14:37588860	Protein coding	—	0.119	0.659	Snapshot	98.73
rs7144658	FOXA1	14:37592537	Protein coding	0.121	0.116	0.940	Snapshot	98.65

^a^MAF: minor allele frequency. ^b^HWE: Hardy-Weinberg equilibrium.

**Table 2:** Distribution of selected demographic variables and risk factors in gastric cancer cases and controls.

	Overall cases (*n* = 577)	Overall controls (*n* = 678)	*P*
	*n* (%)	*n* (%)	
Age (years)	61.34 ± 11.097	62.31 ± 7.549	0.065
Age (years)			
<62	268 (46.45)	324 (47.79)	
≥62	309 (53.55)	354 (52.21)	0.635
Sex			
Male	394 (68.28)	456 (67.26)	
Female	183 (31.72)	222 (32.74)	0.698
Smoking status			
Never	378 (65.51)	493 (72.71)	
Ever	199 (34.49)	185 (27.29)	**0.006**
Alcohol use			
Never	453 (78.51)	520 (76.70)	
Ever	124 (21.49)	158 (23.30)	0.443

**Table 3:** FOXA1 gene rs12894364 polymorphism in GC cases and controls and logistic regression analysis.

Genotype	GC cases (*n* = 577)		Controls (*n* = 678)		Crude OR (95% CI)	*P*	Adjusted OR^a^ (95% CI)	*P*
	*n*	%	*n*	%				
rs12894364								
CC	428	76.29	500	77.40	1.00		1.00	
TC	125	22.28	138	21.36	1.06 (0.80-1.39)	0.686	1.04 (0.79-1.37)	0.796
TT	8	1.43	8	1.24	1.06 (0.81-1.39)	0.649	1.04 (0.63-1.72)	0.874
TC+TT	133	23.71	146	22.60	1.17 (0.44-3.14)	0.758	1.01 (0.88-1.16)	0.858
TT	8	1.43	8	1.24	1.15 (0.43-3.09)	0.776	1.06 (0.65-1.75)	0.810
CC+TC	553	98.57	638	98.76	1.00		1.00	

**Table 4:** FOXA1 gene rs7144658 polymorphism in GC cases and controls and logistic regression analysis.

Genotype	GC cases (*n* = 577)		Controls (*n* = 678)		Crude OR (95% CI)	*P*	Adjusted OR^a^ (95% CI)	*P*
	*n*	%	*n*	%				
rs7144658								
TT	433	77.32	513	78.20	1.00		1.00	
TC	119	21.25	134	20.43	1.05 (0.80-1.39)	0.720	1.07 (0.81-1.42)	0.641
CC	8	1.43	9	1.37	1.05 (0.40-2.75)	0.916	1.04 (0.64-1.69)	0.882
TC+CC	127	22.68	143	21.8	1.05 (0.80-1.38)	0.713	1.03 (0.90-1.19)	0.631
CC	8	1.43	9	1.37	1.04 (0.40-2.72)	0.933	1.03 (0.63-1.67)	0.915
TT+TC	552	98.57	647	98.63	1.00		1.00	

**Table 5:** Analysis of rs7144658 and rs12894364 alleles between cases and controls.

Locus	Variable	Case	Control	*P*	OR (95% CI)
rs12894364	C allele	981 (87.43)	1138 (88.08)		
	T allele	141 (12.57)	154 (11.92)	0.628	0.94 (0.74-1.20)

rs7144658	T allele	985 (87.95)	1160 (88.41)		
	C allele	135 (12.05)	152 (11.59)	0.721	1.05 (0.82-1.34)

**Table 6:** Stratified analyses between rs12894364 polymorphism and risk by sex, age, smoking status, and alcohol consumption.

Variable	Case-control			Adjusted OR (95% CI); *P*				
	CC	TC	TT	CC	TC	TT	(TC+TT) vs. CC	TT vs. (CC+TC)
Sex								
Male	283/334	96/95	5/5	1.00	1.19 (0.86-1.65); *P*: 0.288	1.18 (0.34-4.12); *P*: 0.795	1.19 (0.87-1.64); *P*: 0.280	1.13 (0.33-3.94); *P*: 0.846
Female	145/166	29/43	3/3	1.00	0.77 (0.46-1.30); *P*: 0.330	1.15 (0.23-5.76); *P*: 0.870	0.80 (0.48-1.32); *P*: 0.375	0.95 (0.71-1.29); *P*: 0.754
Age								
<62	202/232	53/63	4/6	1.00	0.97 (0.64-1.46); *P*: 0.870	0.77 (0.21-2.75); *P*: 0.682	0.95 (0.64-1.41); *P*: 0.796	0.99 (0.77-1.28); *P*: 0.955
≥62	226/268	72/75	4/2	1.00	0.97 (0.67-1.41); *P*: 0.866	2.02 (0.37-11.13); *P*: 0.451	0.99 (0.69-1.44); *P*: 0.982	2.30 (0.42-12.67); *P*: 0.324
Smoking status								
Never	290/368	75/95	6/6	1.00	1.00 (0.71-1.41); *P*: 0.992	1.27 (0.41-3.98); *P*: 0.682	1.02 (0.73-1.42); *P*: 0.917	1.27 (0.41-3.97); *P*: 0.682
Ever	138/132	50/43	2/2	1.00	1.11 (0.69-1.78); *P*: 0.659	0.96 (0.13-6.89); *P*: 0.965	1.11 (0.69-1.76); *P*: 0.673	0.93 (0.13-6.68); *P*: 0.943
Alcohol consumption								
Never	340/387	95/102	8/6	1.00	1.06 (0.77-1.45); *P*: 0.717	1.52 (0.52-4.42); *P*: 0.441	1.09 (0.80-1.48); *P*: 0.600	1.50 (0.52-4.35); *P*: 0.454
Ever	88/113	30/36	0/2	1.00	1.07 (0.61-1.87); *P*: 0.812	1.78 (1.57-2.01); *P*: 0.214	1.01 (0.58-1.76); *P*: 0.961	1.79 (1.61-1.99); *P*: 0.210

For FOXA1 gene rs12894364, the genotyping was successful 99.20% in 577 cases and 678 controls.

**Table 7:** Stratified analyses between rs7144658 polymorphism and risk by sex, age, smoking status, and alcohol consumption.

Variable	Case-control			Adjusted OR (95% CI); *P*				
	TT	TC	CC	TT	TC	CC	(TC+CC) vs. TT	CC vs. (TT+TC)
Sex								
Male	291/351	83/83	6/5	1.00	1.21 (0.86-1.70); *P*: 0.282	1.45 (0.44-4.79); *P*: 0.543	1.22 (0.87-1.70); *P*: 0.242	1.39 (0.42-4.60); *P*: 0.585
Female	142/162	36/51	2/4	1.00	0.81 (0.50-1.31); *P*: 0.379	0.57 (0.10-3.16); *P*: 0.520	0.79 (0.49-1.26); *P*: 0.321	0.60 (0.11-3.31); *P*: 0.552
Age								
<62	196/248	57/57	6/4	1.00	1.27 (0.84-1.91); *P*: 0.263	1.90 (0.53-6.82); *P*: 0.318	1.31 (0.88-1.95); *P*: 0.188	1.81 (0.51-6.48); *P*: 0.356
≥62	237/265	62/77	2/5	1.00	0.91 (0.62-1.31); *P*: 0.586	0.45 (0.09-2.32); *P*: 0.326	0.87 (0.60-1.27); *P*: 0.472	0.46 (0.09-2.38); *P*: 0.340
Smoking status								
Never	284/369	82/103	5/6	1.00	1.03 (0.75-1.44); *P*: 0.840	1.08 (0.33-3.58); *P*: 0.896	1.04 (0.75-1.43); *P*: 0.824	1.08 (0.33-3.55); *P*: 0.906
Ever	149/144	37/31	3/3	1.00	1.16 (0.68-1.96); *P*: 0.597	0.97 (1.19-4.87); *P*: 0.967	1.14 (0.68-1.90); *P*: 0.623	0.94 (0.19-4.72); *P*: 0.941
Alcohol consumption								
Never	342/390	93/108	6/6	1.00	0.98 (0.72-1.34); *P*: 0.909	1.14 (0.36-3.60); *P*: 0.821	0.99 (0.73-1.35); *P*: 0.950	1.15 (0.37-3.58); *P*: 0.816
Ever	91/123	26/26	2/3	1.00	1.45 (0.79-2.66); *P*: 0.227	0.97 (1.16-5.90); *P*: 0.971	1.40 (0.78-2.51); *P*: 0.257	0.85 (0.14-5.16); *P*: 0.859

For FOXA1 gene rs7144658, the genotyping was successful 99.60% in 577 cases and 678 controls.

## Data Availability

The data used to support the findings of this study are available from the corresponding authors upon request.
